# Degradation of the Selected Antibiotic in an Aqueous Solution by the Fenton Process: Kinetics, Products and Ecotoxicity

**DOI:** 10.3390/ijms232415676

**Published:** 2022-12-10

**Authors:** Ewa Adamek, Ewa Masternak, Dominika Sapińska, Wojciech Baran

**Affiliations:** Department of General and Analytical Chemistry, Medical University of Silesia, Jagiellońska 4, 41-200 Sosnowiec, Poland

**Keywords:** Fenton reaction, sulfonamides, kinetics, toxicity, degradation products

## Abstract

Sulfonamides used in veterinary medicine can be degraded via the Fenton processes. In the premise, the process should also remove the antimicrobial activity of wastewater containing antibiotics. The kinetics of sulfathiazole degradation and identification of the degradation products were investigated in the experiments. In addition, their toxicity against *Vibrio fischeri*, the MARA^®^ assay, and unselected microorganisms from a wastewater treatment plant and the river was evaluated. It was found that in the Fenton process, the sulfathiazole degradation was described by the following kinetic equation: r_0_ = k C_STZ_^−1 or 0^ C_Fe(II)_^3^ C_H2O2_^0 or 1^ C_TOC_^−2^, where r_0_ is the initial reaction rate, k is the reaction rate constant, C is the concentration of sulfathiazole, Fe(II) ions, hydrogen peroxide and total organic carbon, respectively. The reaction efficiency and the useful pH range (up to pH 5) could be increased by UVa irradiation of the reaction mixture. Eighteen organic degradation products of sulfathiazole were detected and identified, and a possible degradation mechanism was proposed. An increase in the H_2_O_2_ dose, to obtain a high degree of mineralization of sulfonamide, resulted in an increase in the ecotoxicity of the post-reaction mixture.

## 1. Introduction

Sulfonamides (SNs), sulfanilamide derivatives, belong to the most commonly used antibiotics in industrial animal husbandry [1,2,3]. They are chemically stable and are not prone to abiotic hydrolysis, except sulfacetamide [4]. Therefore, when SN residues enter unadopted ecosystems, they can remain there for a long time [2]. Trace amounts of SNs have been detected even in water samples taken from the Arctic regions [5]. 

Even when in low concentrations (below 0.14 µg/kg of soil), SNs are known to disturb the microbial community in the micro-biosphere [6]. This phenomenon can contribute to the development of antibiotic-resistant microorganisms and, potentially, to their transfer and spread to bacterial pathogens [2,3,7,8]. 

Advanced oxidation processes (AOPs) are regarded as effective methods of removing pharmaceutical residues and, simultaneously, hazardous biological materials (including viruses) from wastewater [9,10,11,12,13]. However, a high degree of complexity and a relatively high cost are the major barriers preventing their widespread application [14,15]. 

Many researchers have recognized Fenton and photo-Fenton reactions as one of the most cost-effective AOPs [16,17,18,19,20,21]. Hydroxyl radicals (HO•), being extremely reactive species with a high oxidation potential (E°) of 2.80 V, are generated in these processes [14,15,22,23,24,25,26,27] (Equation (1)):

Fe^2+^ + H_2_O_2_ → Fe^3+^ + HO• + OH^−^ (1)

The supply of HO• radicals can be improved by sunlight irradiation of the reactant solutions [28,29] (Equations (2) and (3)):

Fe^3+^ + H_2_O + hν → Fe^2+^ + HO• + H^+^ (2)

H_2_O_2_ + hν → HO• + H^+^ (3)

During Fenton and photo-Fenton reactions, HO• radicals can react non-selectively with organic compounds [24,26,27,30,31,32,33] (Equations (4)–(6)):

HO• + RH → R• + H_2_O (4)

HO• + R• → ROH(5)

HO• + RH → RHOH(6)

Another known mechanism is the transfer of an electron from an organic compound to the HO• radical, resulting in the formation of a radical cation (RH•^+^) (Equation (7)):

HO• + RH → RH•^+^ + OH^−^ (7)

The resulting organic radicals and organic cation radicals can undergo subsequent reactions (Equation (8)) leading to their complete mineralization:

(8) $$R•\overset{H_{2}O_{2};\;\mathrm{HO}•;{\;O}_{2}}{\to }{\mathrm{CO}}_{2}+H_{2}O+\mathrm{inorganic}\;\mathrm{ions}$$

Reactions in which Fe^2+^ ions are restored can take place simultaneously (Equations (9)–(12)):

Fe^3+^ + H_2_O_2_ ↔ [Fe(HO_2_)]^2+^ + H^+^ (9)

[Fe(HO_2_)]^2+^ + H_2_O_2_ → Fe^2+^ + HO_2_• (10)

Fe^3+^ + HO_2_• → Fe^2+^ + O_2_ + H^+^ (11)

Fe^3+^ + R• → Fe^2+^ + R^+^ (12)

The formed HO• radical can also participate in reactions which are unfavorable when considering the efficiency of organic compound degradation, e.g., (Equations (13) and (14)) [27]:

Fe^2+^ + HO• → Fe^3+^ + OH^−^ (13)

H_2_O_2_ + HO• → H_2_O + HO_2_• (14)

The anions (X^n−^) coordinated with Fe^2+^ ions can actively participate in reactions with HO• [29] (Equation (15)):

HO• + X^n−^ → X•^(n−1)−^ + OH^−^ (15)

These anions may either act as the process activators or inhibitors, depending on the activity of the X•^(n−1)−^ radical [29,34,35,36]. 

The pH value is one of the most important parameters influencing the rate of the Fenton process conducted under homogeneous conditions. The optimal pH falls within the range between 2 and 4 [12,14,15,24,25,37,38,39,40,41,42]. An increase in pH causes the hydrolysis of Fe^2+^ ions, their binding in form of stable complex compounds and increases their oxidation rate in parallel reactions (e.g., with O_2_ dissolved) without the generation of HO• radicals [33]. However, the Fenton process can also be operated at pH > 4, predominantly in the presence of heterogeneous catalysts [15,22,25,38,43]. Some authors have argued that the photo-Fenton process [23,44] or the electron transfer process involving organic ligands and reactive complexes with Fe^(IV)^ [27,45] can effectively occur at a higher pH value.

Other important parameters affecting the efficiency of the Fenton reaction are the reactant concentrations, reaction time, type of oxidized compounds as well as the qualitative composition and charge of pollutants. Even minor deviations from the optimal composition of the reagent concentration may significantly decrease the efficiency of the process [30,44]. 

Another problem during Fenton and photo-Fenton processes is that a high degree of mineralization of organic pollutants requires the use of high doses of reactants. As a result, significant amounts of solid waste will be generated which can be highly ecotoxic. This was confirmed in experiments with *Staphylococcus aureus*, *Escherichia coli*, *Vibrio fischeri* and activated sludge [46,47,48,49]. 

Therefore, if the wastewater treatment process increases the wastewater toxicity, this process should be considered inappropriate and inadequate.

The literature offers abundant information on the application of Fenton and photo-Fenton processes for the degradation of SNs. The researchers have mostly reported reactions carried out in the presence of FeSO_4_ that led to almost 100% drug degradation [15,31,50]. Intermediates most frequently described during these processes include compounds formed as a result of amide hydrolysis and SN derivatives with a hydroxyl group attached to a benzene or heterocyclic ring [24,26,43,51].

The aim of the present study was (1) to determine the empirical kinetic equation of antibiotic degradation in the presence of FeSO_4_/H_2_O_2_ mixture, (2) to compare the efficiency of Fenton and photo-Fenton processes, (3) to identify the degradation products (DPs) of the selected antibiotic by Fenton reagents and (4) to determine the cumulative acute and chronic toxicity of DPs using the commercial tests and microorganisms from the river and effluent (treatment wastewater) from a biological wastewater treatment plant (WWTP). Sulfathiazole (STZ) was chosen as the model antibiotic in the experiments. Explanation of these assumptions is essential from the point of view of Fenton process optimization and estimation of the potential environmental impact of DPs. In our opinion, the experimentally determined kinetic equation may be more universal rather than detailed optimization studies, e.g., due to the variable composition of wastewater. 

## 2. Results and Discussion

### 2.1. Kinetics of STZ Degradation and H_2_O_2_ Decomposition

**Figure 1:**
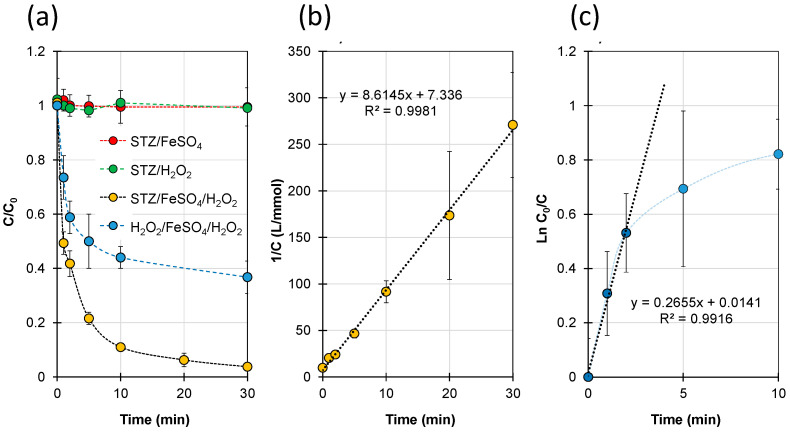
Dynamics of STZ and H_2_O_2_ degradation in the presence of FeSO_4_ and/or H_2_O_2_; (**a**) changes in STZ and H_2_O_2_ concentration during the reaction; (**b**) plot of the function *1/C = f(t)* for change in STZ concentration; (**c**) plot of the function Ln(*C*_0_/*C*) = *f*(*t*) for change in H_2_O_2_ concentration. [FeSO_4_]_0_ = 0.15 mmol/L, [H_2_O_2_]_0_ = 2.0 mmol/L, pH = 3.35–3.45.

Dynamics of STZ (0.1 mmol/L) concentration changes in the presence of FeSO_4_ (0.15 mmol/L) and/or H_2_O_2_ (2.0 mmol/L) at pH 3.35 ± 0.05 are shown in Figure 1a. Dynamics of H_2_O_2_ concentration change in the mixture with STZ and FeSO_4_ were inserted in the same figure. 

In the presence of FeSO_4_ alone or H_2_O_2_ alone and in the absence of light, no changes were observed in the STZ concentration. On the other hand, in a mixture of both reactants, the STZ concentration decreased, indicating the degradation process. For this process, the function of 1/*C = f*(*t*) was found to be a straight line with a high correlation coefficient (R^2^ > 0.99) (Figure 1b). This result indicates that the changes in STZ concentration during the Fenton process followed second-order kinetics. Similarly, second-order kinetics for the degradation of organic compounds during the Fenton process have also been described by other authors [41,45,52,53,54]. 

Initially, the H_2_O_2_ concentration decreased rapidly when a significant excess of H_2_O_2_ with respect to STZ was used in the experiment. Then, the H_2_O_2_ decomposition was inhibited and approximately 40% of this reactant still remained in the mixture after the reaction. The function of Ln(*C*_0_/*C*) = *f*(*t*) for H_2_O_2_ had a linear trend within the first 2 min (Figure 1c). This result indicates that the H_2_O_2_ decomposition followed pseudo-first-order kinetics within this time range. Inhibition of H_2_O_2_ conversion after a longer reaction time may be associated with the oxidation of Fe^2+^ to Fe^3+^. 

### 2.2. Effect of the Initial STZ Concentration

The effect of the initial STZ concentration on the dynamics, efficiency and kinetics of degradation is shown in Figure S1 (Supplementary Materials). The kinetic data were determined experimentally and are shown in Table S1, Supplementary Materials. 

At the lowest STZ concentration (0.05 and 0.1 mmol/L) and after 10 min of the Fenton reaction, its degradation degree was > 90%. The increase in the initial STZ concentration to 1.0 mmol/L led to a lower degree of degradation. The prolongation of the reaction time to 30 min caused an increase in STZ degradation, approximately to 20% (Figure S1b, Supplementary Materials). 

The investigated process showed that an increase in the initial STZ concentration to 0.2 mmol/L caused a proportional decrease in its degradation rate. A further increase in the sulfa-drug concentration (up to 0.2 mmol/L) did not have a significant impact on the reaction rate. Most likely, when the concentration of STZ is higher than that of FeSO_4_, with the excess of H_2_O_2_, the degradation rate is limited by Fe^2+^ ions only. 

Based on the known mechanism of the Fenton reaction, it was expected that the degradation rate would increase as the STZ concentration increased [53]. However, the opposite effect was observed. Other researchers have also described a reduction in the degradation rate of organic compounds linked to an increase in their concentration in the Fenton process [40]. This phenomenon may be associated with a relatively stable binding of Fe^2+^ ions to STZ or its DPs.

**Figure 2:**
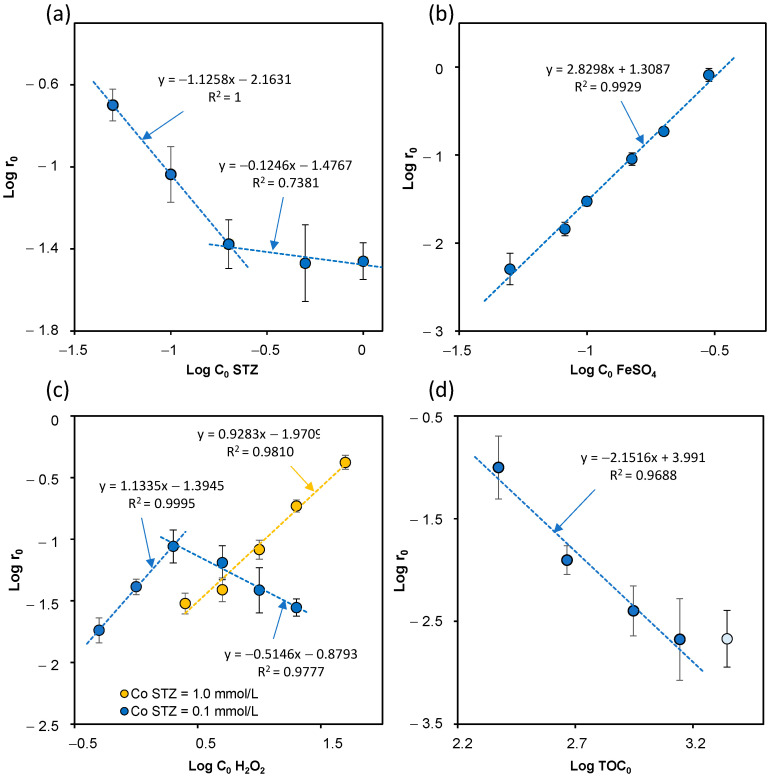
Determination of reaction order; the concentration effect of STZ (**a**), FeSO_4_ (**b**), H_2_O_2_ (**c**) and Total Organic Carbon (TOC) (**d**).

To determine the reaction order with respect to the initial STZ concentration, a Log *r*_0_ vs. Log *C*_0_ plot was constructed for the experimental data (Figure 2a). 

It was found that when *C*_0_ STZ < *C*_0_ FeSO_4_, a linear function with a slope of approximately −1 was obtained. When *C*_0_ STZ > *C*_0_ FeSO_4_, the reaction order with respect to STZ was close to 0. 

Fe^3+^-containing compounds are the most often detected among DPs of Fenton reaction (Equation (8)). These compounds can affect the degradation of SNs in an acidic environment, in the presence of light, microwaves or dissolved oxygen [55]. Additionally, H_2_O_2_ itself can initiate the degradation of SNs and other antibiotics during UV irradiation [29,56]. Our results indicate that under the conditions used, in the absence of light and microwaves, the STZ degradation in the presence of H_2_O_2_ was initiated by Fe^2+^-compounds only. It was not excluded that, in the presence of excess H_2_O_2_, Fe^3+^ ions can be reduced to restore Fe^2+^ ions (Equations (9)–(12)). However, the experimental results indicate that these reactions did not have a significant effect on the rate of STZ degradation.

### 2.3. Effect of the Initial FeSO_4_ Concentration

The results showing the effect of the initial FeSO_4_ concentration (from 0.05 to 0.3 mmol/L) on the kinetics and degradation efficiency of STZ (0.1 mmol/L) in the presence of H_2_O_2_ (2.0 mmol/L) are summarized in Figure S3 and Table S1 (Supplementary Materials). Under the investigated conditions (with a large excess of H_2_O_2_), the increase in FeSO_4_ concentration resulted in a systematic increase in the rate of STZ degradation.

After only 10 min of reaction, it was possible to achieve almost 100% degradation of STZ (Figure S2b), but a minimum of 50% excess of FeSO_4_ with respect to STZ was required in the experiment. However, the prolongation of reaction time to 30 min increased the degree of STZ degradation only by a maximum of 10%. This indicates that organic DPs may effectively compete with STZ molecules and influence the reaction course. 

The described results clearly suggest that the application of a large excess of Fe^2+^ salts can lead to complete drug degradation after a short reaction time. However, this is at the expense of substantial amounts of waste containing Fe^3+^- compounds (mainly hydrated Fe(OH)_3_) formed after the reaction. Strong sorption properties of the hydroxide can improve the total treatment efficiency; nevertheless, with the impurities absorbed, a troublesome and ecotoxic waste is generated.

Figure 2b shows the plot of Log *r*_0_ and Log *C*_0_Fe^2+^ function for the results obtained. In the studied range of FeSO_4_ concentration, the function plot was a straight line with a slope of approximately 3. This result indicates that under the experimental conditions, the initial rate of STZ degradation with respect to the concentration of Fe^2+^ ions could be described by a third-degree equation. However, only some authors have reported such an unusually high order value of the Fenton reaction with respect to the concentration of Fe^2+^ ions [52]. 

The above results show a high influence of the initial FeSO_4_ concentration on the rate and efficiency of the degradation of organic compounds. They also explain the strong inhibition of the Fenton reaction observed when the concentration of Fe^2+^ ions was decreased.

### 2.4. Effect of the Initial H_2_O_2_ Concentration

The effect of the initial H_2_O_2_ concentration on the kinetics and efficiency of STZ degradation was studied at two initial STZ concentrations of 0.1 and 1.0 mmol/L. (Figures S3 and S4, and Table S1, Supplementary Materials). 

At a lower STZ concentration (0.1 mmol/L), an increase in H_2_O_2_ concentration initially resulted in an increase, and subsequently in a reduction in the degree of drug degradation (Figure S3b, Supplementary Materials). An optimal H_2_O_2_ concentration was 2.0 mmol/L; that was when a 20-fold excess of H_2_O_2_ with respect to STZ was used in the experiment (Figure S5, Supplementary Materials). After 30 min, the reaction mixture still contained approximately 10% of STZ and 38% (more than 0.76 mmol/L) of undecomposed H_2_O_2_. Most authors have pointed to 50-fold excess of H_2_O_2_ as an optimal to achieve the complete degradation in the Fenton reaction [53]. However, in our experiment with the solution containing a 100-fold excess of H_2_O_2_, 80% of this reactant was not converted and remained after 30 min of reaction. Additionally, the efficiency of STZ degradation in a 100-fold excess of H_2_O_2_ was much lower. One of the causes of this phenomenon may be a competitive reaction between HO• radicals and H_2_O_2_ [29] (Equation (14)). 

In solutions with a higher initial STZ concentration (1.0 mmol/L), the increase in the H_2_O_2_ concentration resulted exclusively in an increase in the degradation rate (Table S1, Figure S5, Supplementary Materials), an asymptotic increase in the degree of drug degradation, but a decrease in H_2_O_2_ conversion (Figure S4b, Supplementary Materials). Similarly, as observed for the lower STZ concentration, the increase in H_2_O_2_ dose resulted in a lower conversion of this reactant. When a 50-fold excess of H_2_O_2_ to STZ was used, approximately 50% of unreacted H_2_O_2_ remained in the solution after 30 min of reaction. Details on H_2_O_2_ conversion are described in Section 2.8.

The plot of Log *r*_0_ STZ vs. Log *C*_0_ H_2_O_2_ functions for low and high concentration of STZ is presented in Figure 2c. 

Based on the established equations it was found that when [STZ]_0_ = 0.1 mmol/L and [H_2_O_2_]_0_ ≤ 2 mmol/L and when [STZ]_0_ = 1.0 mmol/L in the entire range of H_2_O_2_ concentration, the initial rate of STZ degradation followed first-order kinetics with respect to H_2_O_2_. When [H_2_O_2_]_0_ > 2 mmol/L, its influence on STZ degradation could be determined at the order of approximately −0.5. Many researchers have used the H_2_O_2_:Fe^2+^ molar ratio to characterize the degradation of the reactant during the Fenton process [23,37]. When the molar ratio of H_2_O_2_ varied from 1:1 to 50:1, the degradation rate was proportional to the H_2_O concentration. In turn, when the excess of H_2_O_2_ to Fe^2+^ was >500, the reaction order was (−1) [27]. According to Batista and Nogueira [31], the adverse effect of a significant excess of H_2_O_2_ is also true for the photo-Fenton process.

### 2.5. Effect of the Initial TOC Value

Livestock wastewater mainly contains large amounts of partially decomposed organic matter. These substances react with HO• and iron compounds and may inhibit the Fenton process [12,15,18,23,37,39,57]. Therefore, the dynamics of STZ degradation during the Fenton process were assessed in peptone solutions, simulating wastewater. The organic matter content in these solutions was determined as the TOC value and the results are shown in Figure S6 and Table S1, Supplementary Materials. 

As expected, the increase in the TOC value led to a decrease in the efficiency of STZ degradation. However, even for the highest TOC content (approximately 2200 mg C/L) the degree of STZ degradation was almost 50% (Figure S6b, Supplementary Materials). It was observed that the prolonged reaction time of 10 to 30 min did not significantly improve the efficiency of STZ degradation. The TOC value can also be included in the kinetic equation of STZ degradation. Under the experimental conditions of Fenton process, the reaction order was approximately (−2) (Figure 2d).

It was also confirmed that the removal of TOC from the solution was simultaneous with the STZ degradation during the Fenton process. However, the process efficiency at [TOC]_0_ > 100 mg C/L was lower than that caused by the coagulation and sedimentation of iron compounds (Figure S7, Supplementary Materials). 

### 2.6. Effect of pH

As has already been pointed above, in the homogeneous Fenton process carried out in the presence of Fe^2+^ salts, the optimal pH ranged from 2 to 4. At the higher pH of the solution, Fe^2+^ ions underwent gradual hydrolysis. This result indicates the necessity of acidifying the treated wastewater before adding the Fenton reagents. On the other hand, there are opinions that organic ligands forming coordination compounds with iron ions may also be oxidized at a pH close to neutral [27,45]. SNs form coordination compounds with Fe^2+^ ions [58]. Therefore, it was assumed that the STZ degradation would also be possible at pH values > 4. 

**Figure 3:**
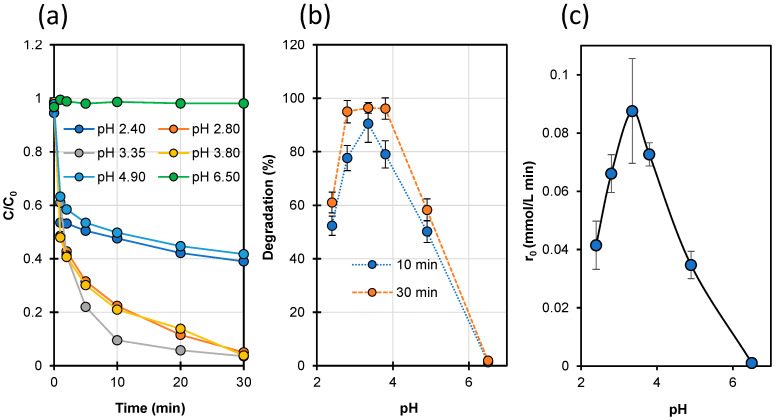
Effect of pH on the dynamics of STZ degradation (**a**), the degree of degradation after 10 and 30 min of reaction (**b**) and the initial degradation rate of STZ (**c**). [STZ]_0_ = 0.1 mmol/L, [FeSO_4_]_0_ = 0.15 mmol/L, [H_2_O_2_]_0_ = 2.0 mmol/L, pH = 3.35 ± 0.05.

The effect of pH on the efficiency of STZ degradation in the Fenton process is illustrated with the data in Table S1, Supplementary Materials and in Figure 3.

It was confirmed that the homogeneous Fenton process was more effective with a pH ranging from 3 to 4. Unfortunately, the STZ degradation was completely inhibited in solutions at pH 6.5 (Figure 3). This may indicate that at a higher pH of solution, complex compounds of Fe^2+^ ions with STZ do not catalyze the conversion of H_2_O_2_ to HO• radicals.

### 2.7. Photo-Fenton Process

**Figure 4:**
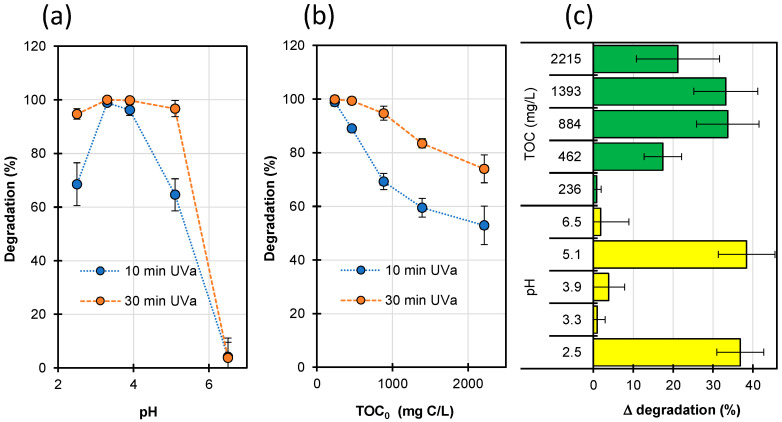
Effect of pH (**a**) and TOC (**b**) on STZ degradation and difference (Δ) in the degree of degradation of Fenton and photo-Fenton processes after 30 min (**c**). [STZ]_0_ = 0.1 mmol/L, [FeSO_4_]_0_ = 0.15 mmol/L, [H_2_O_2_]_0_ = 20.0 mmol/L.

The efficiency of the Fenton process can be increased by irradiating the reaction mixture with UV light (a photo-Fenton process) [23,44]. Therefore, we conducted a series of additional experiments in which the Fenton process was assisted by UVa irradiation. The effect of pH (Figure 4a) and TOC content (Figure 4b) on the efficiency of this process was also studied. Figure 4c shows a comparison of the efficiency for the photo-Fenton and Fenton processes presented as a difference in the degradation degree of after 30 min.

The STZ degradation between 2 and 30 min at pH 2.5 and 5.1 followed zero-order kinetics (Figure S8, Supplementary Materials). This result indicates that under the experimental conditions, the STZ degradation process was light-limited. Moreover, the prolongation of the reaction time resulted in a considerable improvement in the efficiency of the photo-Fenton process (Figure 4a). The effect may be due to the reactions of photoactive Fe^3+^ complexes in the presence of the UVa irradiation [31,33,45,59]. As a result, additional amounts of HO• radicals may be generated, or a charge-transfer process and an oxidation of organic compounds may occur (Equations (16) and (17)):

Fe(HO)^2+^ + hν → Fe^2+^ + HO• (16)

Fe(R)^2+^ + hν → Fe^2+^ + R• (17)

If aliphatic organic acids are present in the solution, they can form complexes with iron ions and H_2_O_2_. In the presence of light, these compounds may also undergo decarboxylation and CO_2_ may be released [60,61]. In the absence of light, the process efficiency depends mainly on Fe^2+^ concentration. As already mentioned (Section 2.1), oxidation of these ions to Fe^3+^ completely inhibits STZ degradation in the Fenton process. 

Use of UVa irradiation allows for a significant increase in photo-Fenton process efficiency in solutions characterized by high TOC content, even by 35% (Figure 4b,c and Figure S9). The degradation efficiency can be even higher when the irradiation time is prolonged. Unfortunately, the cost of wastewater treatment using the photo-Fenton process can be much higher than with application of the Fenton process [14].

### 2.8. Antimicrobial Activity and Ecotoxicity Evaluation

The potential environmental impact of effluent is a key parameter in assessing the efficiency of processes used for wastewater treatment. Liquid waste generated during Fenton and photo-Fenton reactions, which are used to remove antibiotics from wastewater, can contain many substances potentially dangerous to organisms inhabiting a given environment. These compounds could be toxic not only to *S. aureus* and *E. coli*, but also to *Artemia salina*, *Vibrio phosphoreum*, *Dafnia magna* and *V. fischeri* [47,48,49,62,63,64,65].

**Figure 5:**
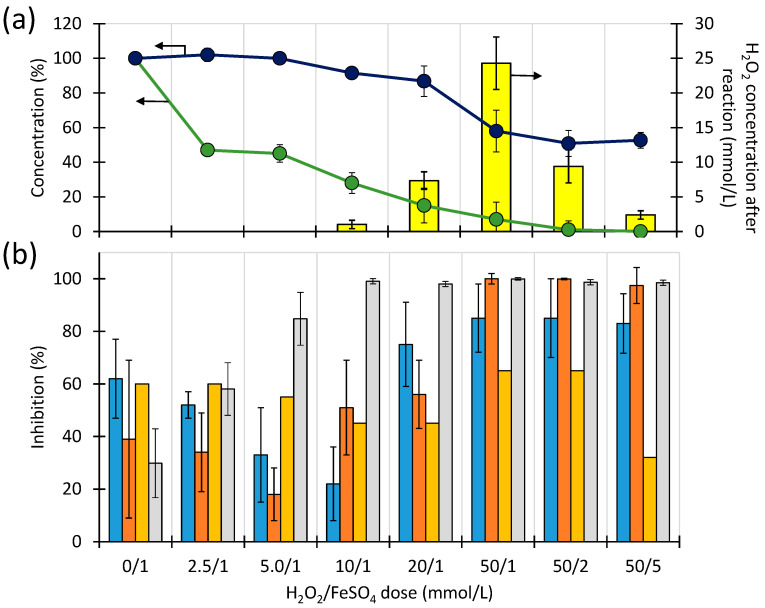
Figure **5.** Effect of H_2_O_2_/FeSO_4_ dose on the relative STZ concentration (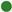), on relative TOC content (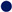) and H_2_O_2_ concentration (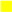) (**a**) and relative inhibition determined for microorganisms from the Brynica River (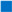), for microorganisms from effluent (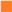), *V fischeri* (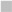), on mean relative inhibition determined for *B. diminuta*, *D. acidovorans* and *P. aurantiaca* (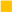) (**b**), in samples after 30 min of Fenton process in the presence of FeSO_4_. [STZ]_0_ = 1.0 mmol/L, pH = 3.35 ± 0.05.

The effect of DPs of STZ (*C*_0_ = 1.0 mmol/L) on the inhibition of *V. fischeri* (Microtox^®^ assay), *B. diminuta*, *D. acidovorans* and *P. aurantiaca* (MARA^®^ assay), unselected microorganisms from the Brynica River (Poland) and from the WWTP effluent is shown in Figure 5b. 

The high initial STZ concentration used in the experiment resulted from a low sensitivity of the studied microorganisms to the antibiotic (Table S4, Supplementary Materials). The results were correlated with the relative concentration of STZ, TOC and with the concentration of H_2_O_2_ remaining in the solution after 30 min of the Fenton process. 

Under the experimental conditions, the increase in H_2_O_2_ and FeSO_4_ dose caused a decrease in the STZ concentration (from 0 to 93%) in solutions after 30 min of the Fenton process. The TOC content decreased by a maximum of 50% indicating that DPs of STZ remained in the solution. Simultaneously, the H_2_O_2_ concentration in the post-reaction solutions increased with increasing the initial dose of this reactant. At a dose of 50 mmol/L, the degree of conversion of H_2_O_2_ was only about 50%. When the FeSO_4_ dose increased from 1 to 5 mmol/L, the degree of H_2_O_2_ conversion increased to 95% (Figure 5a). 

In each of the experiments, the components of the post-reaction solutions were more toxic to *V. fischeri* than the initial STZ solution. The observed inhibition increased to 100% when the remaining H_2_O_2_ concentration increased to approximately 2.5 mmol/L. Therefore, we assume that the inhibitory effect was mainly due to unreacted H_2_O_2_. The toxic effect to river-derived or WWTP-derived microorganisms resulted, on one hand, from STZ itself and, on the other hand, from unreacted H_2_O_2_. As a consequence, the optimal dose range of H_2_O_2_ was between 5 and 10 mmol/L. Changes in STZ-sensitive microorganism inhibition (*B. diminuta*, *D. acidovorans* and *P. aurantiaca)* may be considered as indicators of changes in antibacterial activity resulting from the Fenton process. The reduced STZ concentration caused a decrease in the inhibitory effect against these strains of bacteria. Nevertheless, the unreacted H_2_O_2_ resulted in a toxic effect but weaker than the one discussed above. An increase in FeSO_4_ dose (at constant H_2_O_2_ concentration) decreased inhibition exclusively against *B. diminuta*, *D. acidovorans* and *P. aurantiaca*. The results discussed above indicate that once the Fenton process is applied for wastewater treatment, excess H_2_O_2_ should be avoided. In turn, a higher dose of iron salts will generate significantly higher amounts of waste, e.g., precipitates after neutralization of Fe compounds. Therefore, one main goal should be chosen either to achieve a high degree of mineralization of organic pollutants or to remove their potential ecotoxicity. 

### 2.9. Identification of STZ Degradation Products 

**Figure 6:**
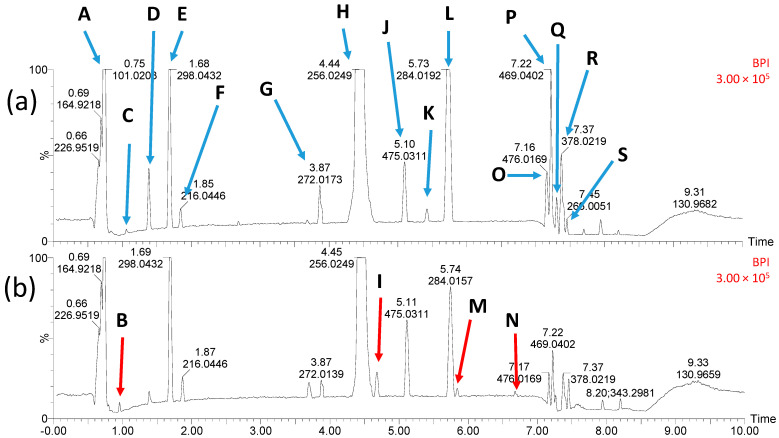
Chromatograms of solutions after STZ degradation with FeSO_4_ and: (**a**) 10 mmol/L H_2_O_2_, (**b**) 50 mmol/L H_2_O_2_. The chromatographic peaks of STZ and DPs are labelled with letters from A to S.

It cannot be excluded that apart from unreacted H_2_O_2_, the DPs with a high toxicity to microorganisms were produced during the Fenton process. As shown in Figure 6, a total of 18 products of the STZ transformation were found in the solution after the Fenton process (STZ is marked on the chromatogram with a letter H). The proposed structural formulas of the identified DPs are included in Figure 6. 

**Figure 7:**
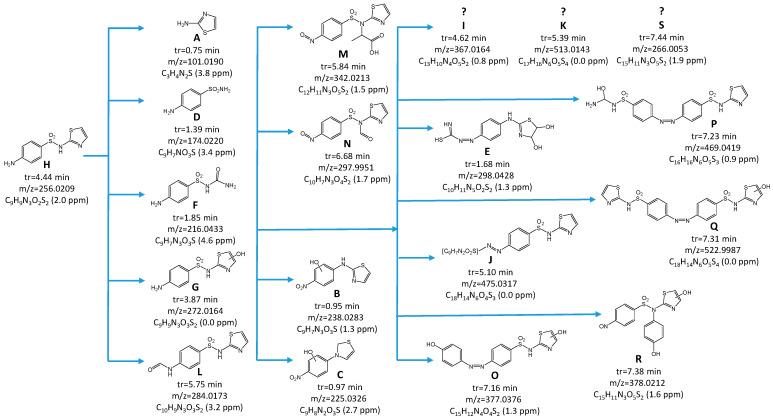
Proposed DPs identified based on the analysis of the chromatograms (Figure 6) and the possible pathway of STZ degradation.

Chromatograms shown in Figure 6a,b correspond to a solution with lower and higher antimicrobial activity and toxicity to environmental microorganisms, respectively. DPs marked with letters B, M, M and N are present on Figure 6b only. This indicates that they were formed in trace amounts in the presence of higher H_2_O_2_ concentration. The MSMS analysis confirmed that the sulfonamide group responsible for the antimicrobial activity of SNs was absent in these products. Therefore, we hold the view that these DPs were not responsible for the increased toxicity of solutions following the Fenton process (Section 2.8). However, DPs marked with letters D, F and G (Figure 7) still had the structure responsible for the antimicrobial activity of SNs. 

The presence of compounds with an attached hydroxyl group among the intermediates indicates the participation of HO• radicals. The radicals may also be responsible for breaking the sulfonamide bond (S single bond N), thus leading to the generation of sulfanilamide and 1,3-thiazol-2-amine as products. The formation of similar DPs has been well-documented to date [24,51,66,67,68,69]. In turn, the addition of oxygen to the amine nitrogen (detected in B, C M, N and R compounds, Figure 7) could in our view result from the red-ox reaction. 

Among the DPs of STZ we also identified the compounds resulting from the elimination of the sulfone group (marked as B, C and E) and previously unknown azo-compounds formed as a result of condensation of STZ with its DPs (marked as O, P and Q). These compounds could arise as a result of termination with the participation of organic radicals or cation radicals. It is not excluded that azo-compounds are formed as a result of charge transfer between organic compounds coordinated with iron. Similar DPs were identified after the photocatalytic degradation of STZ under acidic conditions. Such mechanisms of the Fenton reaction have been described, among others, by Duca et al. [70].

## 3. Materials and Methods

### 3.1. Reactants

A solution of STZ sodium salt (Sigma-Aldrich, St. Louis, MO, USA, >99%, Figure S10) in deionized water (>6 µS/cm) was a main reactant in the experiments. Additionally, FeSO_4_·7H_2_O (Sigma-Aldrich, >99%), H_2_O_2_ (30%, grade for trace analysis, Sigma-Aldrich), H_2_SO_4_ (96%, p.a., Merck, Darmstadt, Germany) and NaOH (p.a., POCH, Gliwice, Poland) were used. An aqueous solution of peptone (peptone from animal proteins, CAS Number: 93384-33-9, Fluka, Buchs, Switzerland) was used in some experiments to simulate a wastewater environment. 

### 3.2. Fenton Process

**Procedure 1**. An aqueous STZ solution (50 mL, 0.05–1.0 mmol/L) was poured into amber glass bottles (100 mL). Then, a calculated volume of FeSO_4_·7H_2_O solution (0.5 mol/L) in H_2_SO_4_ solution (0.05 mol/L) was added. Subsequently, the pH was adjusted with H_2_SO_4_ or NaOH solutions (1.0 mol/L) and an appropriate dose of H_2_O_2_ solution was added. The mixture of reactants was stirred vigorously. All activities were performed in a darkened room, out of direct sunlight, to eliminate the effect of the photo-Fenton reaction.

**Procedure 2**. This procedure was similar to procedure 1, except that an STZ solution (50 mL, 0.1 mmol/L) with peptone (250–400 mg/L) was used.

### 3.3. Photo-Fenton Process

**Procedure 1**. STZ solution (100 mL, 0.1 mmol/L) in deionized water and 30 µL of FeSO_4_·7H_2_O (0.5 mol/L) in H_2_SO_4_ (0.05 mol/L) were stirred in an open crystallizer (500 mL). After adjustment of pH, a H_2_O_2_ solution (30 µL, 3%) was added to the mixture.

**Procedure 2**. This procedure was similar to procedure 1, except that STZ solution (100 mL, 0.1 mmol/L) in peptone solution was used. A volume of added FeSO_4_·7H_2_O was 200 µL and H_2_O_2_ was 300 µL. 

In both procedures, simultaneously with the addition of H_2_O_2_ solution, the mixtures were irradiated with UVa radiation at the intensity of 13.5 W/m^2^. All samples were constantly stirred during irradiation. 

### 3.4. Sampling and Preparation of Samples for Analysis

Before the addition of H_2_O_2_ and after predetermined time intervals, aliquots (5 mL) of the mixtures were collected into ampoules containing NaOH solution (50µL, 1.0 mol/L) and intensively mixed. Then, the ampoules were centrifuged (4000 RPM for 15 min) and the supernatant was immediately analyzed. The final pH of the samples ranged between 6.5 and 8.0.

### 3.5. UPLC Analysis

The analysis was performed using the UPLC technique (ACQUITY UPLC I-Class System, Waters, Milford, MA, USA; column ACQUITY UPLC BEH C18, 130 Å, 1.7 µm, 2.1 mm × 100 mm; detectors: PDA (λ 272 nm) and Xevo G2-XS QTof, ESI+ (Waters). Detailed data for the reagents, the analytical procedures and the mobile phase compositions are shown in Table S2 (Supplementary Materials). 

DPs were identified by comparing the chromatograms using the QTof detector (ESI+) of the samples before and after the addition of H_2_O_2_. If the same monoisotopic molecular masses with the same retention time were recorded on the chromatograms before and after the addition of H_2_O_2_, these compounds were not analyzed. Protonated monoisotopic masses (M + H^+^) were calculated for all DPs of STZ using internal software. Fragmentation spectra of DPs were recorded at collision energy in the range of 10–25 V. The ionization conditions and acquisition parameters for the QToF detector are included in Supplementary Materials (Table S3). The retention time of the standard sulfanilamide was also used to verify its presence in DPs of STZ. All structural formulas were drawn using the ChemDraw Std 15.1 software with the Analysis package (CambridgeSoft, Cambridge, MA, USA). Aliphatic DPs were not recorded or identified on the chromatograms. 

### 3.6. Total Organic Carbon Analysis

The Total Organic Carbon (TOC) analysis was used in the following experiments as a measure of the mineralization of the antibiotic solution. TOC determination was performed using the LCK380 cuvette test (HACH LANGE, Loveland, CO, USA), and the results were read on a DR 3900 spectrophotometer (HACH LANGE). The samples obtained after the degradation processes were diluted prior to the assay using deionized water.

### 3.7. Determination of Residual H_2_O_2_

The concentration of H_2_O_2_ remaining in the neutralized samples was determined using the Quantofix^®^ Peroxyde 25 colorimetric test (Machery-Nagel, Düren, Germany). If necessary, the samples were diluted prior to the assay using deionized water. 

### 3.8. Assessment of Chronic Toxicity

Chronic toxicity of the solution containing STZ (1.0 mmol/L only) and its DPs was evaluated using the MARA^®^ assay (NCIMB Ltd., Aberdeen, Scotland, UK) and unselected microorganisms collected from the water of the Brynica River (50°15′33.1″ N 19°08′12.5″ E; Poland) and from the WWTP effluent (50°18′3.9″ N 19°12′06.0″ E; Poland). In each case, the procedures described by the authors of [71,72] were applied. The details of these experiments are presented in Supplementary Materials. 

### 3.9. Assessment of Acute Toxicity (Microtox^®^ assay)

The acute toxicity of STZ solution (1.0 mmol/L) and DPs formed after 30 min of the Fenton reaction was assessed using the standard Microtox^®^ bioassay with a bioluminescent model bacterium *V. fischeri* [73]. After neutralization by NaOH, the pH of all samples was 7.4 ± 0.6. Bioluminescence inhibition (%) was calculated with the screening test (81.9% basic test protocol) using MicrotoxOmni software (Microtox^®^ Model 500 Analyzer, Modern Water Inc., New Castle, DE, USA). 

The percentage of inhibition versus the control sample that was not exposed to the potential toxicant was measured after a 15-min exposure time.

### 3.10. Calculations

Changes in STZ concentration during the experiments can be described by the following functions (Equations (18)–(20)):

*C*/*C*_0_ = *f*(*t*) (18)

ln *C*_0_/*C* = *f*(*t*) (19)

1/*C* = *f*(*t*) (20)

where *C*_0_ is an initial STZ concentration and *C* is STZ concentration after time (*t*). 

If the result is a linear function characterized by a high correlation coefficient (R^2^) (Figure 1b,c), the conditional degradation rate constant (*k*) and the STZ initial degradation rate (*r*_0_) can be calculated from the slope of the linear function. 

The reaction order of STZ degradation with respect to the reactants used and TOC was determined using a slope of the linear function:

Log *r*_0_ = *f*(Log *C*_0_) (21)

Toxicity effect was defined as a percentage of inhibition (I) according to the equation (Equation (22)):

I = 100 × (*Ec* – *Et*)/*Ec* (22)

where *Ec* is an observed inhibition effect in the control sample and *Et* is an observed inhibition effect in the test sample.

## 4. Conclusions

Under the experimental conditions, the STZ degradation in the Fenton process can be described by the following kinetic equation: *r*_0_ = *k C*_STZ_^−1 or 0^ *C*_Fe(II)_^3^ *C*_H2O2_^0 or 1^ *C*_TOC_^−2^. The initial concentration of FeSO_4_ and the total organic carbon content (TOC) in the solution had a significantly high effect on the rate of STZ degradation.

The reaction proceeded with high efficiency only in an acidic environment in a narrow pH range. Additionally, the STZ degradation required the use of a significant excess of reactants (Fe^2+^ salt and H_2_O_2_) relative to the decomposed substance. The useful pH range and reaction efficiency in solutions with high TOC could be increased by simultaneous UVa irradiation of the reaction mixture (Fe^3+^ compounds formed during the Fenton reaction are likely activated by UVa light). The mineralization of the STZ solution proceeded with an efficiency lower than that of its degradation. Therefore, the stable products of STZ degradation remained in the post-reaction solution. They formed as a result of cleavage of the heterocyclic substituent, hydroxylation, oxidation or condensation of sulfonamide with its DPs. Some DPs demonstrated antimicrobial activity. A higher degree of mineralization required the use of significantly higher doses of Fenton reagents. However, the presence of a large molar excess of H_2_O_2_ led to a partial conversion of this reactant. In our opinion, the undecomposed H_2_O_2_ present among DPs most likely affected the high ecotoxicity of the post-reaction solution. For these reasons, the achievement of a high degree of mineralization in the Fenton or photo-Fenton process is very disadvantageous from the point of view of the amounts and ecotoxicity of wastes. On the other hand, effective antibiotic inactivation can be achieved with only a slight excess of reactants used.

## Data Availability

The data presented in this study are available on request from the corresponding author. The data are not publicity available due to very large sizes of chromatographic files.

## Supplementary Materials

The supporting information can be downloaded at: https://www.mdpi.com/article/10.3390/ijms232415676/s1. References [3,71,74,75,76,77] are cited in the supplementary materials.
