# Supplementary material for: Degradation of the Selected Antibiotic in an Aqueous Solution by the Fenton Process: Kinetics, Products and Ecotoxicity
Source: Int J Mol Sci. 2022 Dec 10;23(24):15676. doi: 10.3390/ijms232415676 (PMC9779365; doi:10.3390/ijms232415676)
Supplement: Supplementary file 1 [file ijms-23-15676-s001.zip › ijms-2064605-supplementary.pdf]

Supplementary Materials

Degradation of the selected antibiotic in aqueous solution by Fenton processes. Kinetics, products, and ecotoxicity

Ewa Adamek, Ewa Masternak, Dominika Sapińska, Wojciech Baran

Table S1. Kinetic data for STZ degradation in the presence of $FeSO_4$ and H_2O_2

C_0 (mmol/L)				pH	k (L/mmol min)	$r_0=k[STZ_0]^2$ (mmol/L min)
STZ	$FeSO_4$	H_2O_2	TOC (mgC/L)			
0.05	0.15	2.0		3.35±0.05	80±10	$2.00±0.25·10^{-1}$
0.1	0.15	2.0		3.35±0.05	9.2±2.0	$9.2±2.0·10^{-2}$
0.2	0.15	2.0		3.35±0.05	1.05±0.20	$4.22±0.81·10^{-2}$
0.5	0.15	2.0		3.35±0.05	$1.40±0.40·10^{-1}$	$3.39±1.02·10^{-2}$
1.0	0.15	2.0		3.35±0.05	$3.47±0.50·10^{-2}$	$3.47±0.50·10^{-2}$
0.1	0.05	2.0		3.35±0.05	$5.07±0.14·10^{-1}$	$5.07±0.14·10^{-3}$
0.1	0.082	2.0		3.35±0.05	1.45±0.18	$1.45±0.18·10^{-2}$
0.1	0.1	2.0		3.35±0.05	2.98±0.26	$2.98±0.26·10^{-2}$
0.1	0.15	2.0		3.35±0.05	9.05±1.04	$9.05±1.04·10^{-2}$
0.1	0.2	2.0		3.35±0.05	18.6±0.6	$1.86±0.06·10^{-1}$
0.1	0.3	2.0		3.35±0.05	81.5±10.0	8.15±1.00
0.1	0.15	0.5		3.35±0.05	1.82±0.60	$1.82±0.60·10^{-2}$
0.1	0.15	1.0		3.35±0.05	4.11±0.42	$4.11±0.42·10^{-2}$
0.1	0.15	2.0		3.35±0.05	8.76±2.82	$8.76±2.82·10^{-2}$
0.1	0.15	5.0		3.35±0.05	6.45±1.43	$6.45±1.43·10^{-2}$
0.1	0.15	10		3.35±0.05	3.89±1.12	$3.89±1.12·10^{-2}$
0.1	0.15	20		3.35±0.05	2.79±0.32	$2.79±0.32·10^{-2}$
1.0	1.0	2.5		3.35±0.05	$3.69±0.41·10^{-2}$	$3.69±0.41·10^{-2}$
1.0	1.0	5		3.35±0.05	$3.89±0.60·10^{-2}$	$3.89±0.60·10^{-2}$
1.0	1.0	10		3.35±0.05	$8.24±1.03·10^{-2}$	$8.24±1.03·10^{-2}$
1.0	1.0	20		3.35±0.05	$1.86±0.15·10^{-1}$	$1.86±0.15·10^{-1}$
1.0	1.0	50		3.35±0.05	$4.20±0.12·10^{-1}$	$4.20±0.12·10^{-1}$
0.1	0.15	2.0		2.4±0.05	4.15±0.83	$4.15±0.83·10^{-2}$
0.1	0.15	2.0		2.8±0.05	6.61±0.65	$6.61±0.65·10^{-2}$
0.1	0.15	2.0		3.35±0.05	8.76±1.82	$8.76±1.82·10^{-2}$
0.1	0.15	2.0		3.8±0.05	7.27±0.40	$7.27±0.40·10^{-2}$
0.1	0.15	2.0		4.9±0.05	3.47±0.47	$3.47±0.47·10^{-2}$
0.1	0.15	2.0		6.5±0.05	$1.08±0.91·10^{-1}$	$1.08±0.91·10^{-3}$

0.1	1.0	20	236±4	3.35±0.05	9.96±4.60	$9.96 \pm 4.60 \cdot 10^{-2}$
0.1	1.0	20	462±12	3.35±0.05	1.25±0.28	$1.25 \pm 0.28 \cdot 10^{-2}$
0.1	1.0	20	884±20	3.35±0.05	$3.79 \pm 0.15 \cdot 10^{-1}$	$3.79 \pm 0.15 \cdot 10^{-3}$
0.1	1.0	20	1393±10	3.35±0.05	$2.11 \pm 0.12 \cdot 10^{-1}$	$2.11 \pm 0.12 \cdot 10^{-3}$
0.1	1.0	20	2215±20	3.35±0.05	$2.14 \pm 0.09 \cdot 10^{-1}$	$2.14 \pm 0.09 \cdot 10^{-3}$

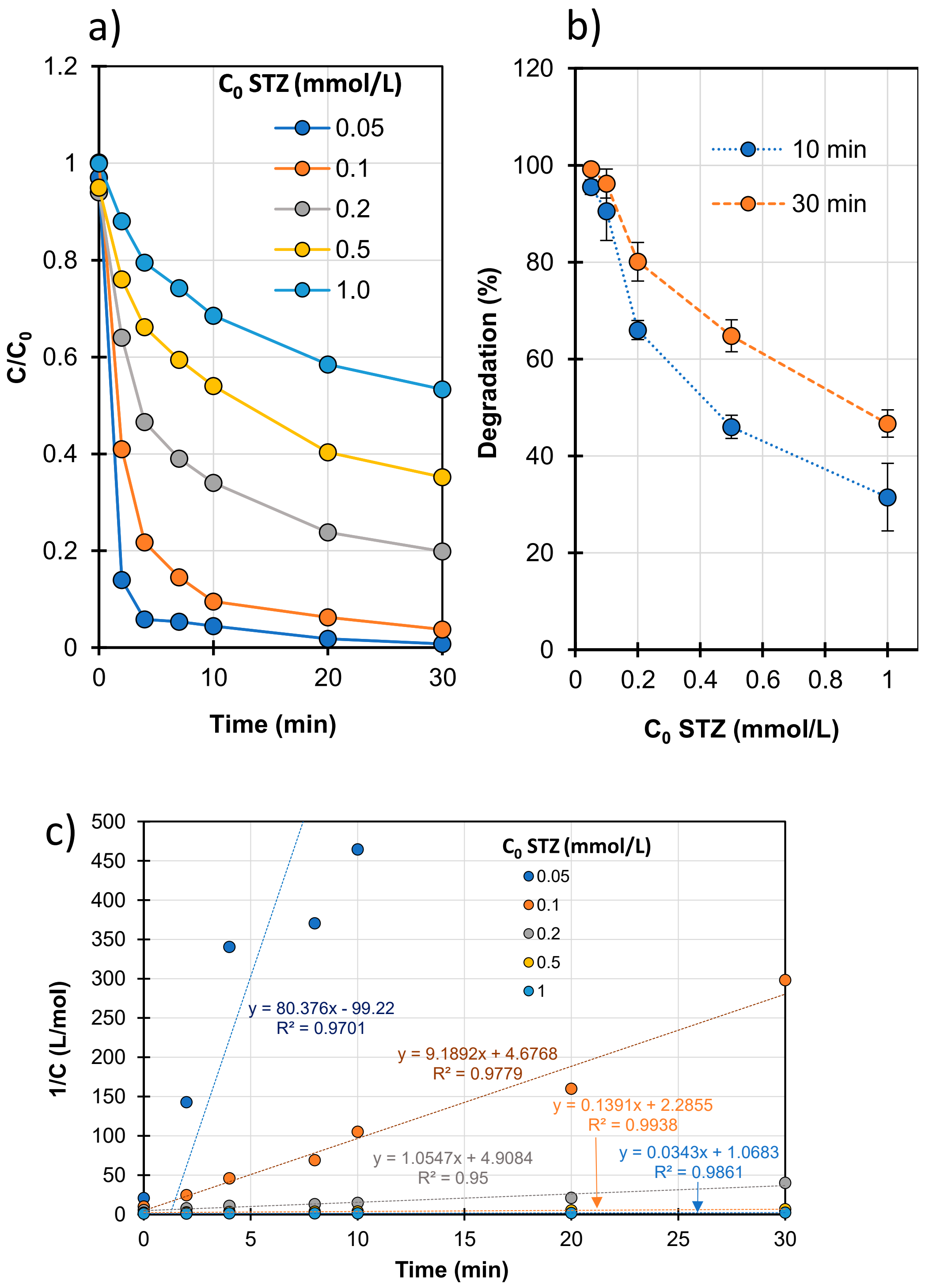

Figure S1. The effect of STZ concentration on the dynamics of degradation (a), the degree of STZ degradation after 10 and 30 minutes of reaction (b) and plots, equations and R^2 values of the linear functions: *1/C = f(t)* (c). $[FeSO_4]_0$ = 0.15 mmol/L, $[H_2O_2]_0$ = 2.0 mmol/L, pH= 3.35±0.05.

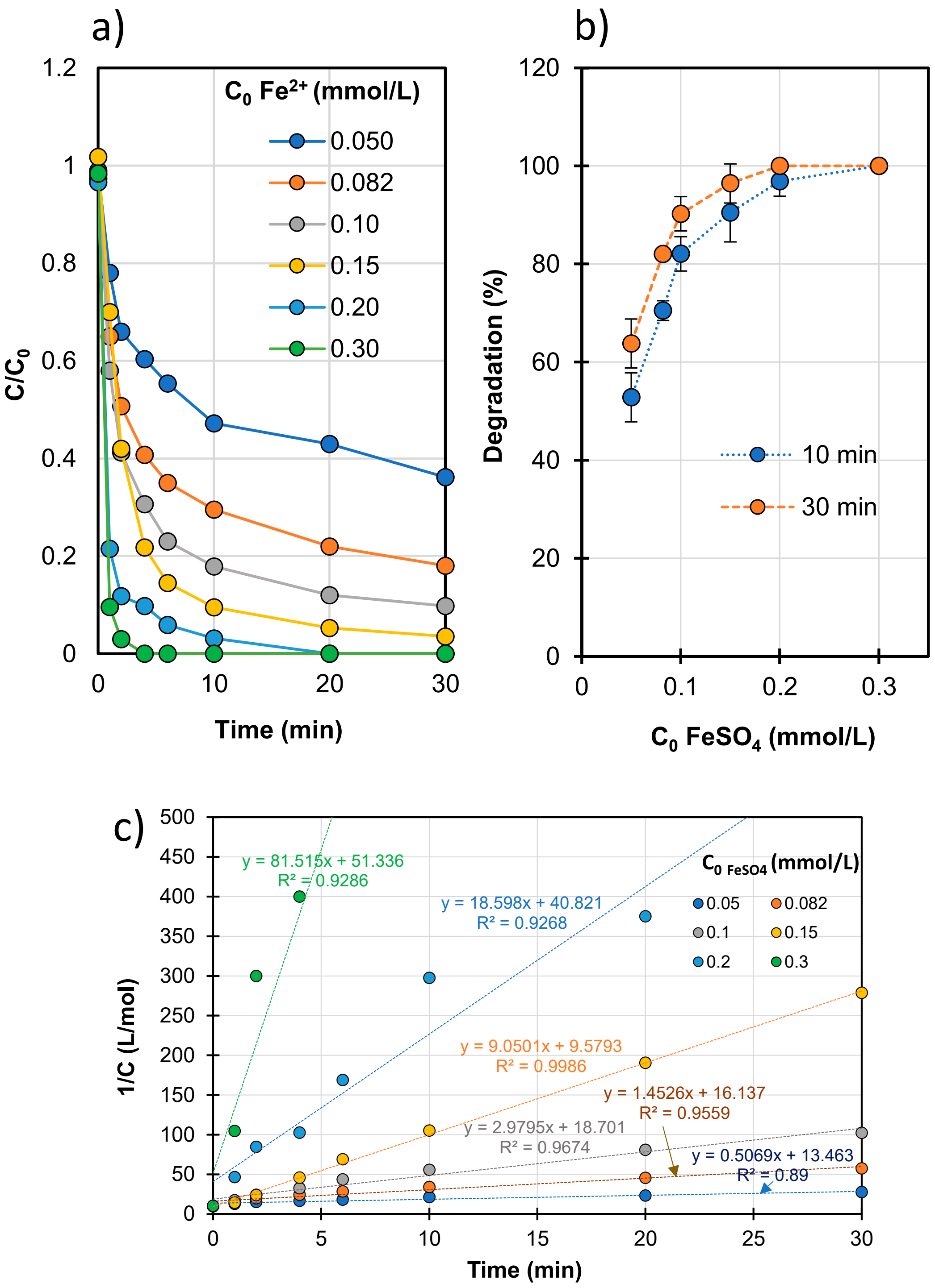

Figure S2. Effect of $FeSO_4$ concentration on the dynamics of STZ degradation (a), the degree of STZ degradation after 10 and 30 minutes of reaction (b), and plots, equations, and R^2 values of the linear functions *1/C = f(t)* (c). $[STZ]_0 = 0.1$ mmol/L, $[H_2O_2]_0 = 2.0$ mmol/L, pH= 3.35±0.05

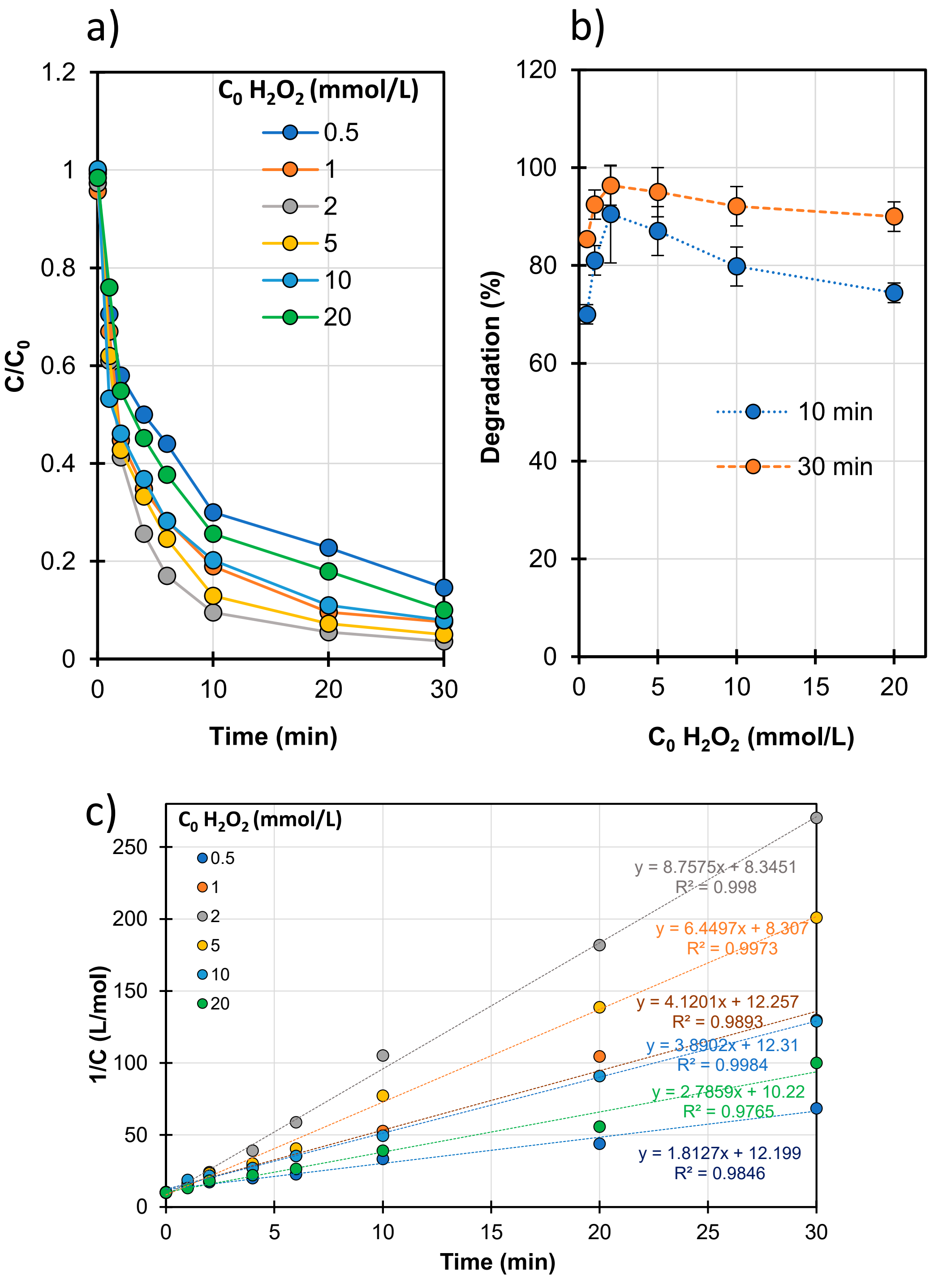

Figure S3. Effect of H_2O_2 concentration on the dynamics of STZ degradation (a), the degree of STZ degradation after 10 and 30 minutes of reaction (b), and plots, equations and R^2 values of the linear functions: *1/C = f(t)* (c). $[STZ]_0$ = 0.1 mmol/L, $[FeSO_4]_0$ = 0.15mmol/L, pH= 3.35±0.05.

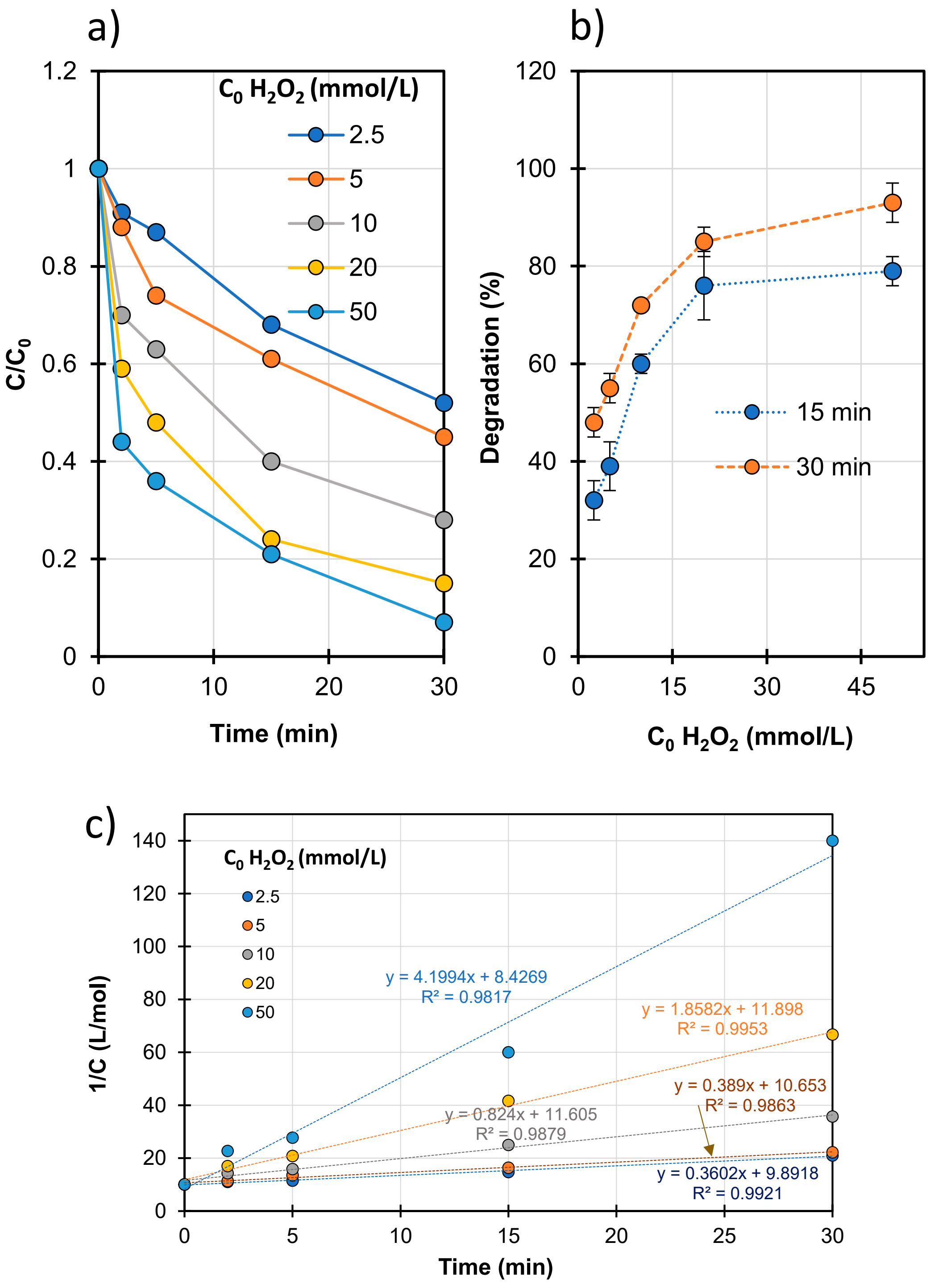

Figure S4. Effect of H_2O_2 concentration on the dynamics of STZ degradation (a), the degree of STZ degradation after 10 and 30 minutes of reaction (b), and plots, equations and R^2 values of the linear functions: *1/C = f(t)* (c). $[STZ]_0 = 1.0$ mmol/L, $[FeSO_4]_0 = 0.15$mmol/L, pH= 3.35±0.05

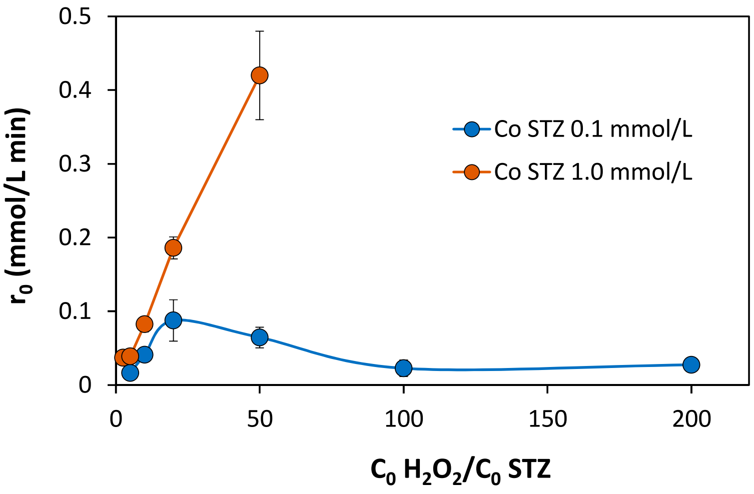

Figure S5. Effect of the concentration ratio of H_2O_2 to STZ on the degradation rate of STZ.

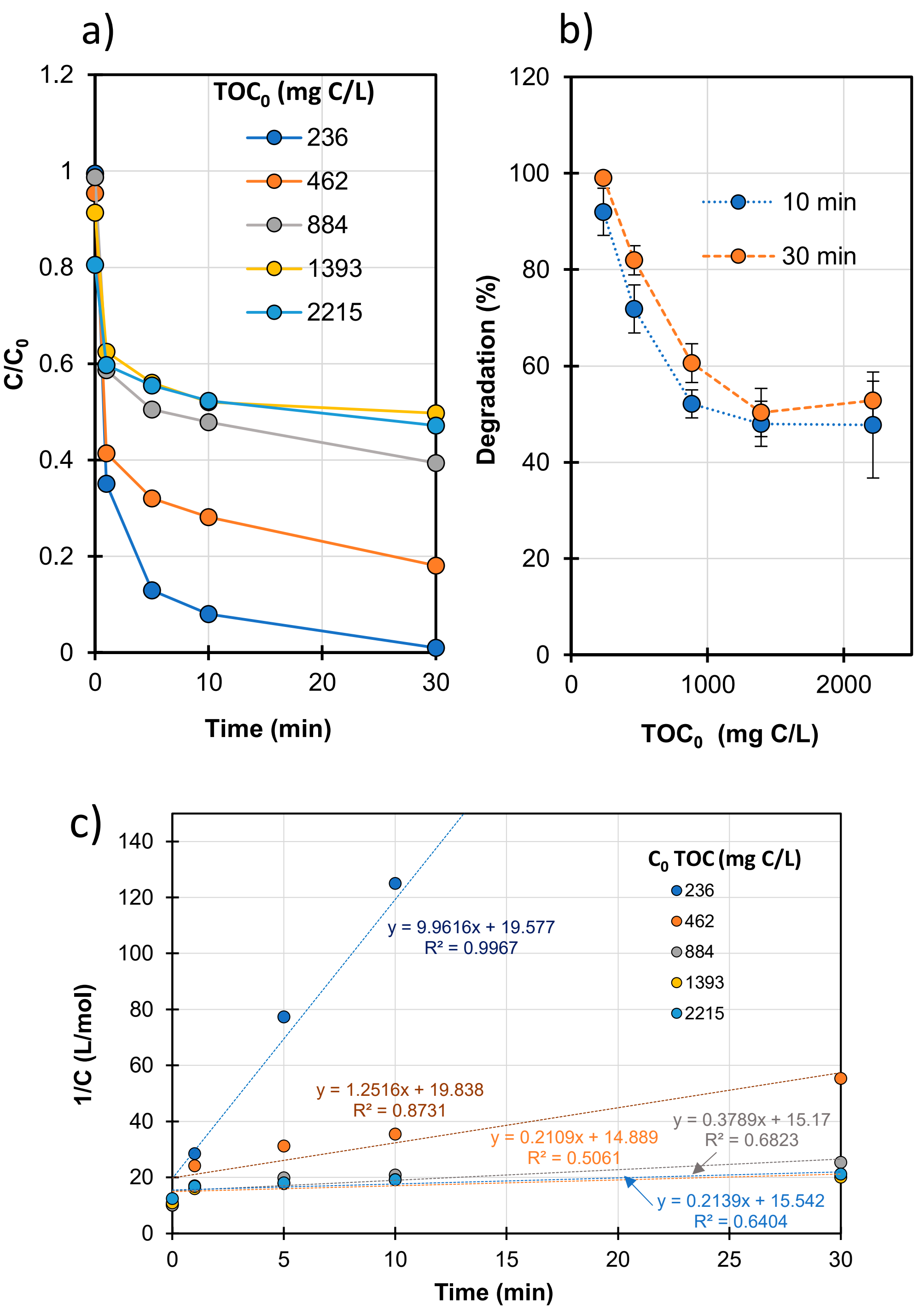

Figure S6. Effect of TOC on the dynamics of STZ degradation degradation (a), the degree of STZ degradation after 10 and 30 minutes of reaction (b) and plots, equations and R^2 values of linear the functions: *1/C = f(t)* (c). $[STZ]_0 = 0.1$ mmol/L, $[FeSO_4]_0 = 1.0$ mmol/L, $[H_2O_2]_0 = 20.0$ mmol/L, pH = 3.20±0.10

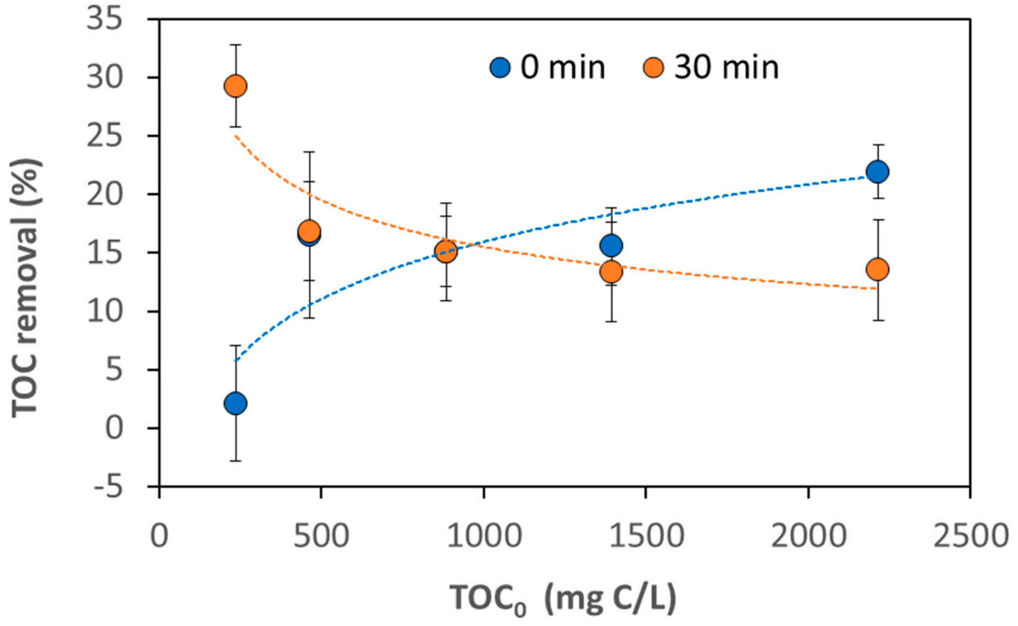

Figure S7. Effect of TOC value of STZ (0.1 mmol/L) and peptone solution on the TOC removal after 30 min of Fenton reaction and after coagulation (precipitation of Fe hydroxides). $[FeSO_4]_0$ =1.0 mmol/L, $[H_2O_2]_0$ = 20 mmol/L, pH = 3.1-3.3

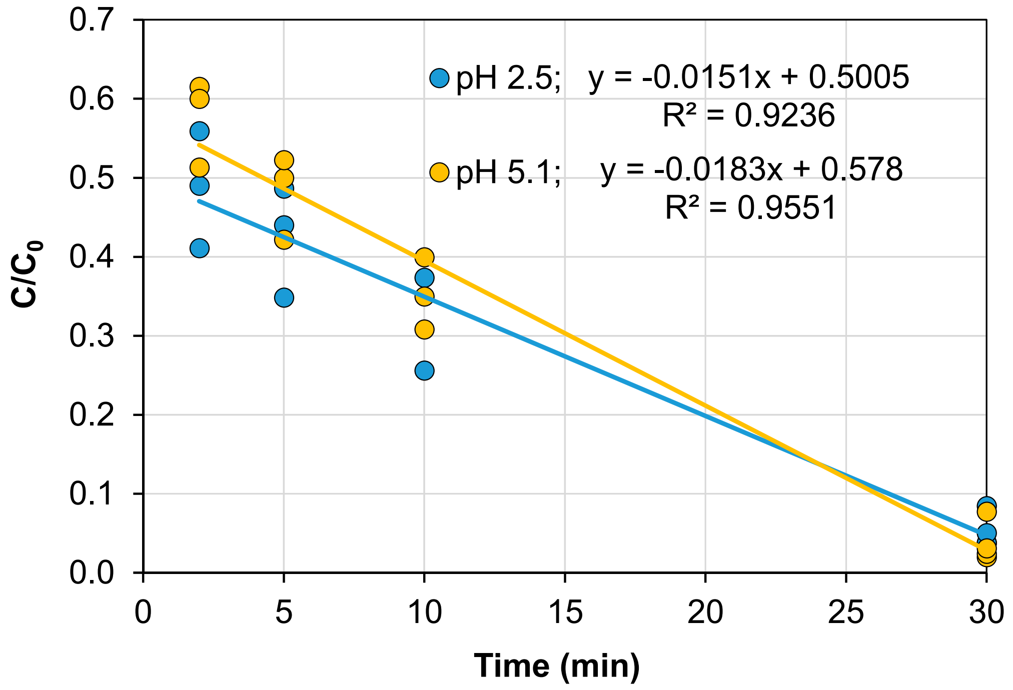

Figure S8. Plot of the function $C/C_0 = f(t)$ for the photo-Fenton reaction at pH 2.5 and 5.1

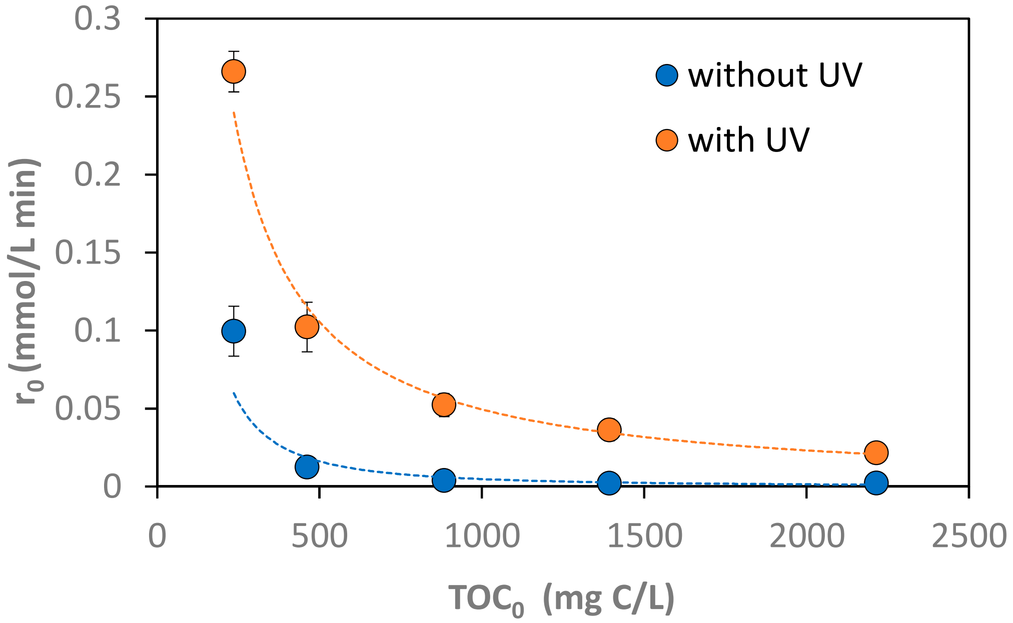

Figure S9. Effect of TOC value of peptone solution on the initial degradation rate of STZ (0.1 mmol/L) during Fenton and photo-Fenton reactions. $[FeSO_4]_0$ =1.0 mmol/L, $[H_2O_2]_0$ = 20 mmol/L, pH = 3.1-3.3

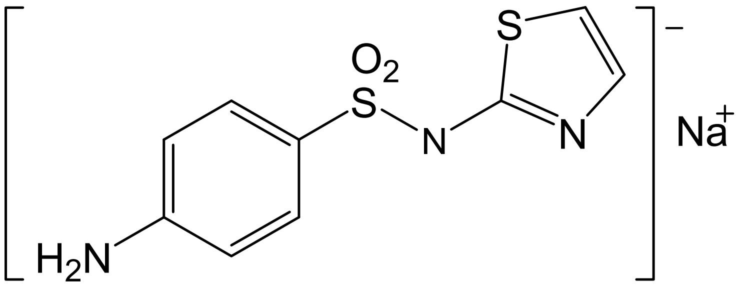

Figure S10. Structure of sulfathiazole sodium salt

Table S2. Data of the experimental conditions (reagents used, analytical procedures, and mobile phase composition)

Column temperature - 35ºC		
Sample volume – 1 μL and 5 μL		
Mobile phase A: H_2O (LC-MS grade, LiChrosolv® Sigma-Aldrich) with 0.01% HCOOH (98% - 100% for LC-MS LiChropur®, Supelco)		
Mobile phase B: CH_3CN (hypergrade for LC-MS LiChrosolv® Sigma-Aldrich) with 0.01% HCOOH		
Gradient		
Time (min)	A (%)	B (%)
0	95	5
3.5	80	20
6.5	50	50
7.5	50	50
8.3	95	5
10	95	5

Table S3. Ionisation conditions and acquisition parameters for the Xevo Qtof detector (XEVO G2 XS) created by Masslynx v4.1

Experimental Instrument Parameters	
Polarity	ES+
Analyser	Resolution Mode
Capillary (kV)	3.0000
Sampling Cone	40.0000
Source Temperature (°C)	100
Source Offset	80
Desolvation Temperature (°C)	250
Cone Gas Flow (L/Hr)	50.0
Desolvation Gas Flow (L/Hr)	600.0
LM Resolution	4.7
HM Resolution	15.0
Aperture 1	0.0
Pre-filter	2.0
Ion Energy	0.2
Manual Collision Energy	FALSE

Collision Energy	6.0
Detector	2825
DetectorCache	0
Sample Infusion Flow Rate (µL/min)	25
Sample Flow State	LC
Acquisition mass range	
Start mass	50.000
End mass	600.000
Scan Time (sec)	0.500
Interscan Time (sec)	0.014
Set Mass	Manual From Chromatogram
Start Mass	50.0
MSMS End Mass	600.0
Start Time (mins)	0.00
End Time (mins)	10.00
Data Format	Continuum
ADC Sample Frequency (GHz)	6.0
ADC Pusher Frequency (µs)	60.0
ADC Pusher Width (µs)	1.50
Use Tune Page Cone Voltage	YES
Use Auto Collision Energy	NO
Collision Energy (eV)	10.0-25.0
Sensitivity	Normal
Dynamic Range	Normal
Calibration	Dynamic 2

1. Assessment of the ecotoxicity of STZ solution

To assess the toxicity of STZ solution to microorganisms, the MARA® bioassay (NCIMB Ltd, Scotland) was used in the experiments. This assay consists of microorganisms belonging to taxonomically diverse groups and having different sensitivities to various toxic substances (Table S4) [74]. The MARA® assay can be successfully used to test a toxicity of antibacterial drugs [75,76]. Additionally, the results using this biotest can be regarded as more representative and reliable than those obtained in single-species tests [76]. The indicator microorganisms were incubated in phytone peptone (2% w/w, Becton, Dickinson & Co.) medium. The growth of test micro-organisms was observed as a change in colour of 2,3,5-triphenyltetrazolium chloride (TZR, p.a.; POCH; Poland). Growth of the tested micro-organisms resulted in pellet formation in the wells with a pellet size proportional to the degree of growth [71,74,75]. Microbial Toxic Concentration (MTC; Eq. S1) was determined using the MARA® software (NCIMB Ltd, Scotland).

$$MTC = C_{\min} \times d^{(P_{tot}/P_0)-1} \quad \text{(S1)}$$

where C_{min} is the lowest concentration in the gradient, d is the dilution factor, P_o is the pellet size in the control well and P_{tot} is the sum of the pellet sizes in all wells that were exposed to the concentration gradient of the antibiotic solutions. Each test was performed in triplicate.

The initial STZ concentration used for the determination of MTC was 300 mg/L. Details of the procedure used in the assay are described in section 3 and shown in Figure S11a.

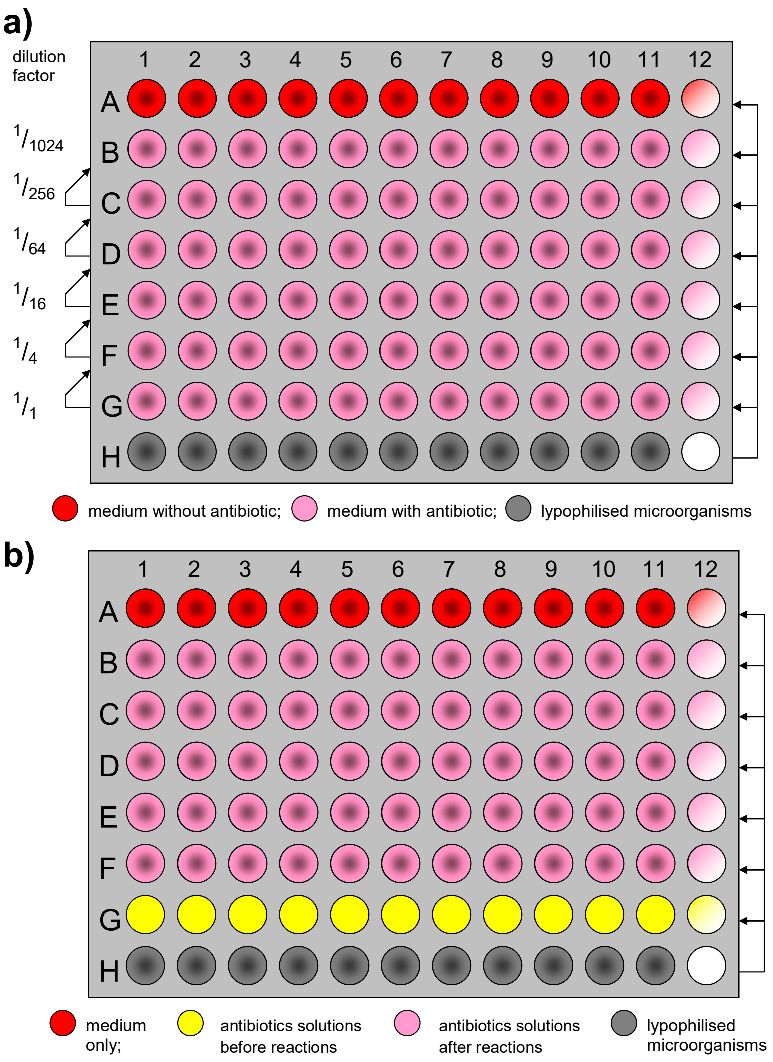

Figure S11. Standard procedure for preparing plates a) to assess the STZ toxicity (determination of MTC values) and b) to determine the antimicrobial activity of STZ solutions after the Fenton process.

The changes in each plate were recorded after 48 hours of incubation and analysed using the MARA® software. The results are expressed as means (n=3) and standard deviations of the MTC values (Table S4).

Table S4. Microbial Toxic Concentration (MTC) values of the STZ and the test microorganisms after 48 hours of incubation [3]

Species		MTC (mg/L)
1.	*Microbacterium* spp.	29.5±6.5
2.	*B. diminuta*	9.4±1.1
3.	*C. freundii*	275±41
4.	*C. testosterone*	240±55
5.	*E. casseliflavus*	> 300
6.	*D. acidovorans*	28.5±0.5
7.	*K. gibsonii*	221±0
8.	*S. warneri*	213±34
9.	*P. aurantiaca*	125±22
10.	*S. rubidaea*	218±24
11.	*P. anomalia*	> 300

2. Determination of the antimicrobial activity of STZ solutions before- and after Fenton process

The antimicrobial activities of the solutions containing STZ and its DPs were also assessed using the MARA® assay. After the end of the Fenton process, a solid phytone peptone (0.4 g) and 200 µL of TZR solution (1%) were added to 10 mL aliquots of the solutions. Subsequently, each sample was filter sterilized (sterile syringe filters CA 0.20, LLG GmbH). After filtration, acidic samples were neutralized with NaOH solution under sterile conditions. Additionally, the initial STZ solution with the addition of $FeSO_4$ was prepared and served as a negative control. Sterilized aliquots were applied directly to the microplates as shown in the Figure 11Sb. Example scans of test plates obtained in the experiments are presented in Figure S12.

a) b)

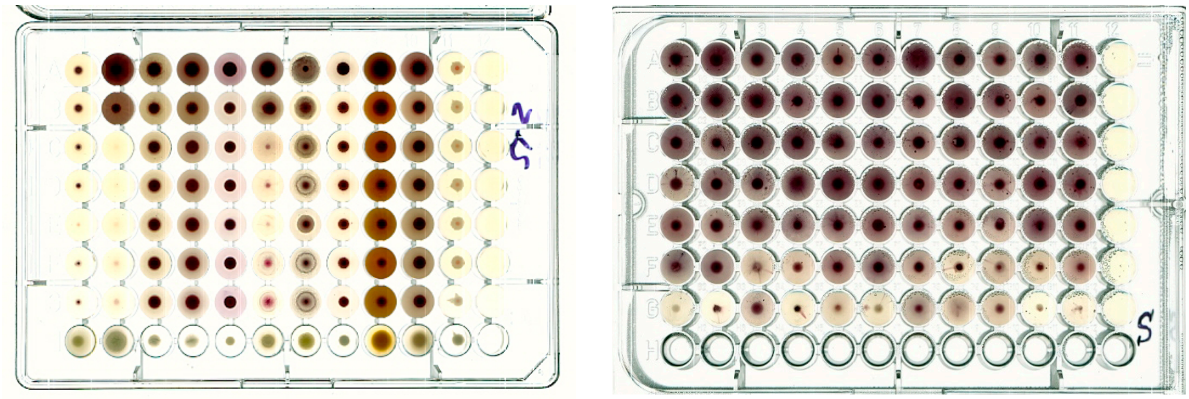

Figure S12. Example scans of test plates a) MARA® bioassay after incubation with STZ solution, b) DPs of STZ after incubation with effluent from WWTP

3. Procedure used in the bioassays

In row H of MARA® assay, columns 1-11 contain lyophilized species of 1-*Microbacterium* spp., 2-*B. diminuta,* 3-*C. freundii,* 4-*C. testosterone,* 5-*E. casseliflavus,* 6-*D. acidovorans,* 7-*K. gibsonii,* 8-*S. warneri,* 9-*P. aurantiaca,* 10-*S. rubidaea* and 11-*P. anomalia* [77]. Column 12 is a negative control for the biotest (no microbes). Initially, 150 µL of the sterile microbial growth medium (Phytone Peptone; Becton, Dickinson, & Co. San Jose, CA, US) at a concentration of 2% (w/w) was added to each well in row H. Microplates were incubated for 4 h at 303 K. After incubation, 150 µL of sterile growth medium (2% Phytone Peptone with 0.01% TZR) was added to each well in rows A-F. Additionally, 200 µl of sterile solution of STZ in Phytone Peptone (2%) with 0.01% TZR was added in row G. 50 µL of aliquots were transferred from each well in row G to the corresponding wells in row F. After mixing of the well contents, 50 µL of sample from each well in row F was transferred to wells in row E. This procedure was repeated for row B. Row A contained 150 µL of sterile medium only, without antibiotic solutions (positive control). Finally, to inoculate the STZ solutions with the bacterial suspension, 15 µL of each microbe sample was transferred from the wells in row H to the corresponding wells in row G. This process was repeated for rows A through F. Microplates were incubated for 48 h at 303 K.

For effluent from WWTP and Brynica river, the procedure was similar except that the wells were inoculated with these samples.

Samples after Fenton process were applied to the plates but without their dilution (Figure S11b)
